# Extraction of Flavonoids from the Flowers of *Abelmoschus manihot* (L.) Medic by Modified Supercritical CO_2_ Extraction and Determination of Antioxidant and Anti-Adipogenic Activity

**DOI:** 10.3390/molecules21070810

**Published:** 2016-06-25

**Authors:** Jingjing Li, Juan Zhang, Min Wang

**Affiliations:** 1 School of Life Science and Technology, China Pharmaceutical University, #24 Tong Jia Xiang, Nanjing 210009, China; pingljj@126.com; 2 Department of Pharmaceutical Engineering, Zhejiang Pharmaceutical College, #888 Yin Xian Avenue Eastern Section, Ningbo 315000, China

**Keywords:** *Abelmoschus manihot* (L.) Medic, flavonoid, modified supercritical CO_2_, antioxidant activity, anti-adipogenic activity

## Abstract

*Abelmoschus manihot* (L.) Medic has been used for many years in Chinese traditional medicine. In this study, supercritical CO_2_ plus a modifier was utilized to extract flavonoids from the flowers of *Abelmoschus manihot* (L.) Medic. The effects of temperature (40 °C–60 °C), pressure (10–30 MPa) and different concentrations of ethanol as modifier (60%–90%, ethanol:water, *v*/*v*) on major flavonol content and the antioxidant activity of the extracts were studied by response surface methodology (RSM) using a Box-Behnken design. The flavonol content was calculated as the sum of the concentrations of seven major flavonoids, namely rutin, hyperin, isoquercetin, hibifolin, myricetin, quercetin-3′-*O*-glucoside and quercetin, which were simultaneously determined by a HPLC method. The antioxidant activity was evaluated by a 2,2-diphenyl-1-picrylhydarzyl (DPPH) free radical-scavenging assay. The results showed that three factors and their interactions could be well fitted to second-order polynomial models (*p* < 0.05). At the optimal extraction conditions for flavonol content (20 MPa, 52 °C, and 85% ethanol content), the yield of flavonoids was 41.96 mg/g and the IC_50_ value was 0.288 mg/mL, respectively, suggesting the extract has high antioxidant activity. Furthermore, the anti-adipogenic activity of the extract on the 3T3-L1 cell line was investigated. The results indicated that it can downregulate PPARγ and C/EBPα expression at mRNA. In summary, in this study, we have established a cost-effective method for the extraction of flavonoids from the flowers of *Abelmoschus manihot* (L.) Medic using supercritical fluid extraction and the extracts exhibited potent antioxidant and anti-adipogenic effects, suggesting a possible therapeutic approach for the prevention and treatment of obesity.

## 1. Introduction

**Figure 1:**
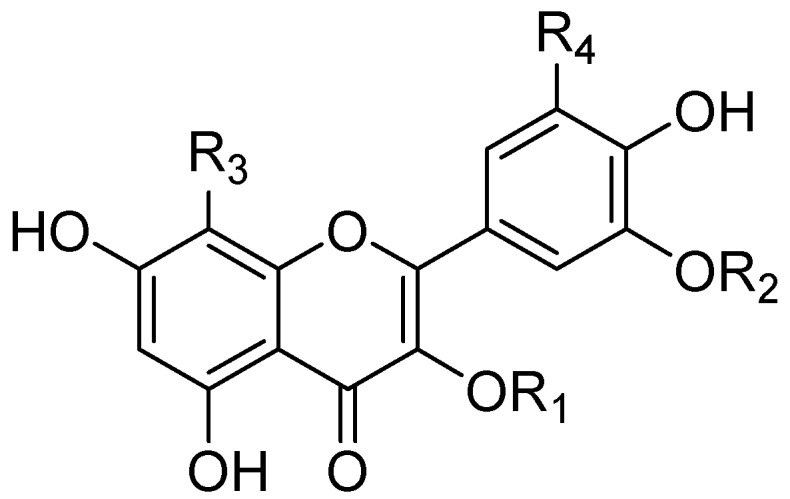
The structures of the seven flavonoids.

*Abelmoschus manihot* (L.) Medic is widely used in China. The flowers are an important herbal medicine for the treatment of chronic renal disease [1,2], diabetic nephropathy [3], oral ulcers [4] and burns. In recent years, the structure of its seven main flavonoids were identified as follows: rutin, hyperin, isoquercetin, hibifolin, myricetin, quercetin-3′-*O*-glucoside and quercetin [5]. Their structures are shown in Figure 1. At present, the standard of quality for *Abelmoschus manihot* in the China Pharmacopoeia (2015 Edition) is a content of hyperin of no less than 0.5% [6]. However, hyperin is not present in sufficient quantities to determine the flavonol content in the flowers of *Abelmoschus manihot*. In this study, the major flavonol content, measured as the sum of the contents of seven major pharmacological flavonoids by simultaneous HPLC determination was used as the detection index to explore the extraction process of flavonoids from the flowers of *Abelmoschus manihot*.

**Compound****R_1_****R_2_****R_3_****R_4_**Rutinrha→gal (β-1,6)HHHHyperingalHHHIsoquercetinglcHHHHibifolinHH*O*-glcHMyricetinHHHOHQuercetin-3′-*O*-glucosideHglcHHQuercetinHHHH

Several techniques for the extraction of flavonoid components from herbal medicines have been studied, such as reflux extraction [7], microwave-assisted extraction [8], and ultrasound extraction [9]. With the renewed attention to the environment, an excellent extractive technique should be efficient, clean and environmentally friendly. In this regard, supercritical CO_2_ extraction is a perfect green technique owing to the facts it is fast, cheap and toxic solvent-free [10]. However, supercritical CO_2_ extraction is only suitable in non-polar compounds. When it is used to extract polar compounds such as flavonoids, a polar solvent is required as a modifier in the supercritical CO_2_. There are no reports using supercritical fluid extraction (SFE) for flavonoids from the flowers of *A. manihot*.

In the human body free radical oxidation is related to human aging and many diseases, so people are increasingly concerned about antioxidant levels. The antioxidant properties of flavonoids have been studied [11], but less attention has been paid to the extracts obtained with SFE, especially from *A. manihot*. Meanwhile, methods for antioxidant activity evaluation are receiving more and more attention, and the generally used methods are the Ferric Reducing Antioxidant Power (FRAP), 2,2-azino-bis(3-ethylbenzothiazoline-6-sulfonic acid (ABTS), Trolox Equivalent Antioxidant Capacity (TEAC) and 2,2-diphenyl-1-picrylhydarzyl (DPPH) ones [12]. Compared with the other methods, the DPPH method is a sensitive, rapid and accurate assay for evaluating the activity of antioxidants [13]. 

Obesity as a key risk factor for ill-health cannot be ignored, because it can cause type II diabetes, hypertension, coronary heart disease, and cancer [14]. Obesity is characterized by an excessive accumulation of fat cells in the human body [15]. It can be regulated by the differentiation of adipocytes and inhibition adipogensis from preadipocytes to adipocytes [16]. Adipocyte differentiation is a highly regulated process, in which many kinds of transcription factors, signaling pathways and miRNA are required [15]. Among the various types of transcription factors, peroxisome proliferator-activated receptor-γ (PPARγ) and CCAAT/enhancer binding protein α (C/EBPα) play an important role in adipocyte differentiation [17], which can upregulate the expression of enzymes and functional proteins which are related to lipid accumulation and lipid metabolism.

In this study, we investigated: (i) the effects of SFE extraction parameters including pressure, temperature and ethanol concentration in the yield of major flavonol and antioxidant activity using a response surface methodology based on a Box-Behnken experimental design; (ii) the relationship between the flavonol content and antioxidant activity under the optimum condition; (iii) the effects of extracts on PPARγ and C/EBPα expression at mRNA using the 3T3-L1 cell line.

## 2. Results

### 2.1. Single Factor Level Experiments

**Figure 2:**
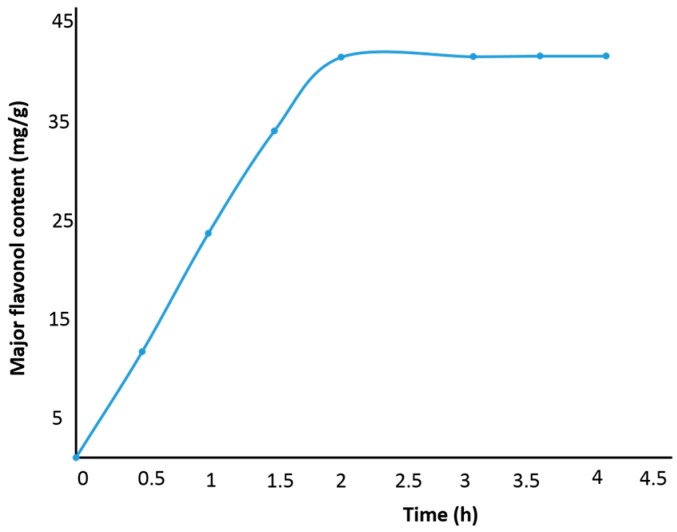
The effect of extraction time on the major flavonol yield.

Before the experimental design experiments were performed, rational ranges for the variables of the model were selected by single-factor experiments (see Supplementary Materials, Figures S1–S3). For this it was considered that the extraction yield is mainly influenced by factors such as extraction temperature, pressure, modifier content and time. The effect of extraction time on the major flavonol yield is shown in Figure 2. As seen in the figure, it is possible to recover >95% of the major flavonoids within 2 h at a temperature of 50 °C, pressure of 20 MPa and with an ethanol content of 80%. Therefore, extraction time was not taken as a variable and a value of 2 h was used. The ranges of the other factors like temperature, pressure and ethanol content were determined and then the effects of the variables together and their interactions were evaluated in the subsequent Box-Behnken experimental design. 

### 2.2. Analysis of Response Surfaces

Response surface optimization has advantages compared to traditional single variable optimization. It saves time, raw material and can evaluate the interactions of the different variables. The quadratic model from the Box-Behnken design can be used to generate a response surface image for the main interactions among extraction pressure (X_1_), temperature (X_2_) and ethanol content (X_3_). The statistical analysis of the quadratic models based on ANOVA is shown in Table 1 and Table 2. The results indicated that the proposed models were significant with *p*-value < 0.0001 and 0.0012, the coefficient of determination (R^2^) were 0.9892 and 0.9456, and the adjusted coefficients of determination (Adj. R^2^) were 0.9753 and 0.8756, respectively. Meanwhile, the lack of fit were not significant (*p* > 0.05). The models were reasonable to predict major flavonol yield and antioxidant activity. 

The response surface Equations (1) and (2) are obtained to predict Y_1_ and Y_2_, respectively:
Y_1_ = 40.35 + 0.47X_1_ + 1.49X_2_ + 2.71X_3_ − 0.27X_1_X_2_ − 0.21X_1_X_3_ + 0.59X_2_X_3_ − 2.97X_1_^2^ − (1)2.93X_2_^2^ − 0.91X_3_^2^Y_2_ = 0.30 + 0.034X_1_ − 0.001X_2_ − 0.047X_3_ + 0.016X_1_X_2_ − 0.031X_1_X_3_ + 0.011 X_2_X_3_ + 0.016X_1_^2^ (2)+ 0.025X_2_^2^ + 0.030X_3_^2^


Equation (1) implies that increasing the pressure (X_1_), temperature (X_2_) and ethanol content (X_3_) can increase the major flavonol content (Y_1_). The interaction term X_2_X_3_ had a positive effect while X_1_X_2_ and X_1_X_3_ had a negative effect on the major flavonol content (Y_1_). Equation (2) implies that temperature (X_2_), ethanol content (X_3_) and the interaction term X_1_X_3_ have a synergistic effect on the antioxidant activity, yet pressure (X_1_) and interaction term X_1_X_2_, X_2_X_3_ did the opposite.

**Figure 3:**
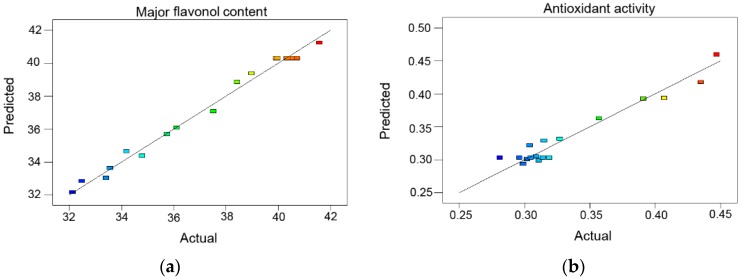
Correlation graph between the predicted and experimental yield values. (**a**) The correlation graph of major flavonol content; (**b**) The correlation graph of antioxidant activity.

Correlation graphs showed that a high correlation existed between the experimental and predicted values. Figure 3 indicates the good fit of both models as illustrated by the fact each point is close to the corresponding regression line. Based on Equations (1) and (2), the optimal extraction conditions were 19.81 MPa, 52.47 °C and 84.92% ethanol solution with a maximum yield of 41.96 mg/g major flavonoids; and 10.15 MPa, 49.86 °C and 85.29% ethanol solution gave the minimum IC_50_ value of 0.281 mg/mL.

### 2.3. Effect of Extraction Parameters

**Figure 4:**
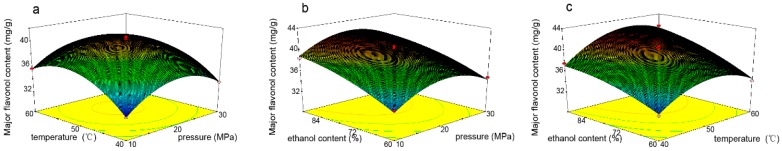
Response surface and contour plots of major flavonol content showing: (**a**) the effect of pressure and temperature at constant 75% ethanol concentration; (**b**) the effect of pressure and ethanol content at constant temperature 50 °C; (**c**) the effect of temperature and ethanol content at constant pressure 20 Mpa.

**Figure 5:**
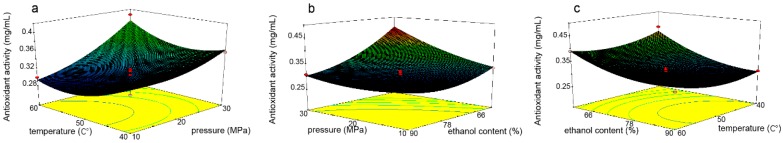
Response surface and contour plots of antioxidant activity showing (**a**) the effect of pressure and temperature at constant 75% ethanol concentration; (**b**) the effect of pressure and ethanol content at constant temperature 50 °C; (**c**) the effect of temperature and ethanol content at constant pressure 20 Mpa.

3D response surface plots can be used to illustrate the interaction effect between any two variables while the other one is held at a constant optimum level. The relationships between the dependent variables (the flavonol content and IC_50_ value) and three factors (pressure, temperature and ethanol content) are shown in Figure 4 and Figure 5.

The interaction effect between pressure and temperature (X_1_X_2_) on each dependent variable is shown in Figure 4a and Figure 5a, while ethanol content (X_3_) is kept at a middle value of 75%. It was observed that higher major flavonol yield (>40 mg/g) was attained when the pressure was set between 18 MPa to 25 MPa and temperature was between 48 °C to 57 °C. Further increases in pressure and temperature actually lowered the yield. Increased solubility with pressure is due to an increase in the density of CO_2_, and decreased solubility with pressure due to decreased solvation capacity [18]. The solubility influenced by temperature is also balanced by two opposing factors: solute vapor pressure and solvent density [19,20]. For the DPPH radical-scavenging activity of the extracts, a lower value of IC_50_ (<0.300 mg/mL) occurred at pressures below 18 MPa and temperatures above 45 °C. However, temperature had no significant effect on both dependent variables (*p* > 0.05).

The effects of the combination of pressure and ethanol content are shown in Figure 4b and Figure 5b. Higher major flavonol content (>40.0 mg/g) were obtained at pressures between 18 MPa and 23 MPa and ethanol concentrations above 80% (Figure 4b). A high concentration range of ethanol/water can increase flavonol content due to a similar polar solvent dissolving a similar polar solute. However, higher ethanol concentration (>90%) would decrease the extraction rate with the increase of fat soluble substances. Lower IC_50_ values (<0.300 mg/mL) of extracts were obtained when the pressure was less than 20 MPa and ethanol concentration was from 75% to 85%. The interaction between pressure and ethanol content was significant (*p* < 0.05) for both models.

When considering the effects of temperature and ethanol content on the flavonol content, higher yields were obtained at higher temperature and higher ethanol content (Figure 4c). As ethanol content increased further, the yield decreased due to repulsive solute-solvent interactions. For the DPPH radical-scavenging activity of the extracts, smaller values of IC_50_ (<0.300 mg/mL) were attained when the ethanol content was set above 78% and the temperature below 55 °C (Figure 5c).

### 2.4. The Relationship Between Major Flavonol Content and IC_50_ Value

**Figure 6:**
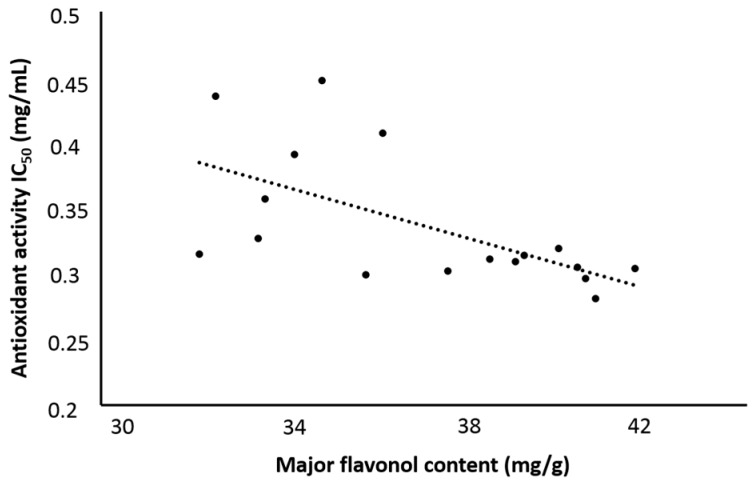
The correlation between major flavonol content and antioxidant of extracts.

A negative correlation (r = −0.611) between the major flavonol content and IC_50_ value of extracts is shown in Figure 6. It meant that the extracts with higher flavonoids content usually had smaller IC_50_ values (higher antioxidant activity). 

### 2.5. Comparison Between Major Flavonol Content and IC_50_ Value of Extracts with Two Methods

In order to validate the optimal supercritical extraction conditions, a verification experiment was carried out. The predictive values are compared with experimental ones which were obtained with the predicted optimized conditions yielding the highest flavonol content, and integral values of the parameters were taken as follows: 20 MPa, 53 °C, and 85% ethanol content. Experimental values were no significantly different from the predicted values within the 95% confidence interval (Table 3). Furthermore, in comparison with Soxhlet extraction, there is also no significant difference between two extraction methods (Table 3). Considering the shorter extraction time, less organic solvent consumption and lower environmental pollution, SFE is an efficient technique for the exhaustive extraction of the flowers of *A. manihot*.

### 2.6. Effect of Different Concentrations of Extracts on the Anti-Adipogenic Activity

**Figure 7:**
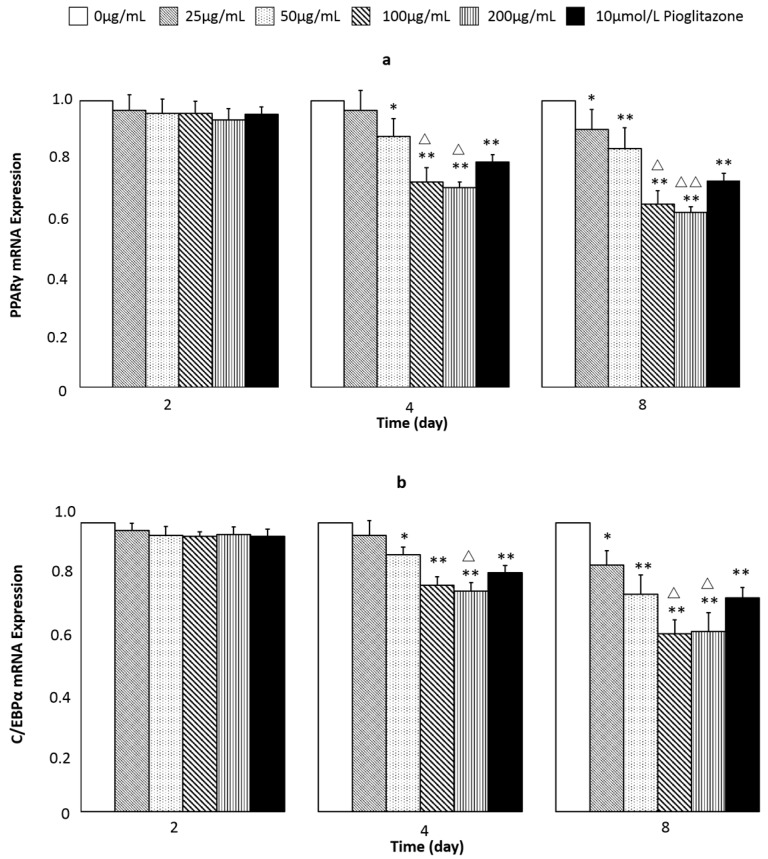
Effect of different concentrations of extracts on the mRNA expression of PPARγ and C/EBPα in 3T3-L1 cells in the 2 days, 4 days and 8 days. The results were determined by RT-PCR. Data are represented as mean ± SD. *n* = 3. * *p* < 0.05, ** *p* < 0.01, compared with control. ^△^ *p* < 0.05, ^△△^ *p* < 0.01, compared with pioglitazone.

To evaluate the inhibitory mechanism of extracts (obtained under the optimum conditions) during the adipocyte-differentiation period, the expression of PPARγ and C/EBPα as transcription factors were examined at 2, 4 and 8 days. The results showed that the expression of both genes remained at a low level in the early stage (2 days) of the differentiation period (Figure 7a). As the process went on, PPARγ expression levels gradually increased in a time-dependent manner. On day 8, the different dosage groups and the pioglitazone group levels were significantly lower than those of the normal groups. It was noted that 100 μg/mL dosage group had a notable difference compared with pioglitazone group. The inhibitory expression of C/EBPα reached a peak in the 8th day (Figure 7b). The highest value was 38.33% in 100 μg/mL dosage which showed a remarkable decrease compared to the pioglitazone group. These findings verified the inhibitory action of extracts on the differentiation of 3T3-L1 preadipocytes.

## 3. Discussion

With the economic development of society and the resulting different lifestyles, obesity has become one of the most common nutritional imbalance disorders. More and more people suffer from obesity, and research into natural anti-obesity products has become a hot topic in recent years [21]. For a long time, SFE technology was mainly used to extract nonpolar substances because supercritical CO_2_ has low polarity. A certain concentration of co-solvent is necessary to improve the separation efficiency of flavonoids using supercritical CO_2_ extraction. SFE is influenced by many extraction parameters, including pressure, temperature, volume of co-solvent, extraction time, modifier constituent and so on. Traditional experiment designs change one variable at a time. However it is hard to estimate the relationship between the variables. In this study, three main influencing factors were screened out by previous single-factor experiments, then a Box-Behnken experimental design with three variables at three levels was used, which allows us to determinate the interactions among the factors and improve the quality of prediction [22]. According to ANOVA, pressure and ethanol content had significant effects on the major flavonol content and antioxidant activity. It was believed that the solubility of flavonoids increased at a given concentration range of ethanol/water, which could be explained by the fact of a similar polar solvent dissolving a similar polar solute. However, excessive ethanol concentration would decrease extraction rates with the increase of liposoluble components. Pressure was also in favor of the extraction of flavonoids, but further increases in pressure didn’t improve the yield due to the repulsive solvent interaction. The optimum experimental conditions predicted by the mathematical models was pressure 20 MPa, temperature 53 °C and ethanol content 85%. The results of the prediction agree well with the actual values. Furthermore, there is no significant difference between SFE and Soxhlet extraction methods. It is thus revealed that SFE is an efficient technique for flavonoid extraction from the flowers of *A. manihot*. This is the first study to analyze extraction efficiency by the sum of the contents of seven major pharmacological flavonoids in the flower of *Abelmoschus manihot*.

It is believed that flavonoids are the primary contributors to the antioxidant activity of the flowers. However, the experimental results showed the maximum flavonol yield didn’t correspond to the minimum IC_50_ value, which means there are some other components contributing the antioxidant activity (Figure 6). Based on the above analysis, further research to look for new plant-based antioxidant compounds is warranted. 

3T3-L1 is recognized as a model for the study of adipocyte differentiation [23]. After treatment with the differentiation mixture culture (containing insulin, DEX, IBMX), 3T3-L1 preadipocytes progress to adipocytes. Two important transcription factors include CCAAT/enhancer binding protein family (C/EBPs) and peroxisome proliferators-activated receptor family (PPARs) were found in the differentiation process [23].

It is found that C/EBPs, including C/EBP-α, β and δ, are important transcription factors in adipocyte differentiation [24]. Research on the tumor suppressor p53 shows that the expression of its downstream genes can be activated while binding with C/EBPα, thus blocking cell proliferation and turning to differentiation [25]. C/EBPβ can be bound to the binding sites of many gene promoters to regulate their expression [26]. C/EBPδ, as a transcriptional activator, takes part in adipocyte differentiation in the early stage [27]. PPARs control many cellular metabolism. PPARγ, as an important member of the family, is responsible for cell differentiation and transcription. A study found that PPARγ-deficient preadipocyte can’t be induced into adipocytes with any factor’s stimulation [28]. When PPARγ the plasmid was transfected into 3T3-L1 preadipocytes, the cells can spontaneously differentiate into adipocytes [29]. It indicates that PPARγ is a necessary factor for fat formation.

During the process of in vitro cell culture, the expression of C/EBPβ and C/EBPδ is transiently induced in early stage of differentiation. Subsequently, the expression of PPARγ and C/EBPα is induced, which activate a large number of downstream target gene expressions. Finally, preadipocytes differentiate into adipocytes [30]. PPARγ and C/EBPα play essential roles in the differentiation progress, as both of them are key adipocyte marker genes on the adipocyte differentiation of 3T3-L1 cells [23]. Therefore, to investigate the effects of extracts on the adipogenesis, 3T3-L1 preadipocytes were treated with various concentrations of extracts during differentiation. Our data showed that the extracts significantly attenuated the expression of PPARγ and C/EBPα compared with adipocytes, revealing that the extracts inhibited adipogenesis in the 3T3-L1 cells by down-regulating PPARγ and C/EBPα expression. 

Based on above analysis, we believe extracts from SFE with noticeable antioxidant and anti-adipogenic activity have potential to become a drug or health care product for the prevention and treatment of obesity.

## 4. Materials and Methods

### 4.1. Materials

The flowers of *A. manihot* were obtained from Suzhong Pharmacy (Taizhou, China). The flowers were ground using an herbal pulverizer (FW 100, Tianjin Taisite Instrument Co. Ltd., Tianjin, China) and sieved to obtain the particles smaller than 0.3 mm. Rutin, hyperin, isoquercetin, myricetin, and quercetin standards were purchased from the National Institutes for Food and Drug Control (Beijing, China). Hibifolin and quercetin-3′-*O*-glucoside were made in the lab. DPPH was supplied by Sigma-Aldrich Co. Ltd. (Shanghai, China). The mouse embryo 3T3-L1 cell line was obtained from the American Type Culture Collection (Manassas, VA, USA) and cryopreserved at the Department of Biochemistry of China Pharmaceutical University. Dulbecco′s modified eagle medium (DMEM) and fetal bovine serum (FBS) were purchased from JianCheng Bioengineering Institute (Nanjing, China). Insulin, dexamethasone (DEX), dimethyl sulfoxide (DMSO), 3-isobutyl-1-methylxanthine (IBMX) and Rneasy MiNi Kit was purchased from QIAGEN (Germantown, MD, USA). RT PrimeScript Kit and SYBR Premix Ex Taq were purchased from TaKaRa (Dalian, China). CO_2_ (Fangxin Gas Ltd., Ningbo, China, purity 99.5%) was used in all extraction experiments. Pioglitazone was purchased from Takeda Co. (Tokyo, Japan). All other solvents were analytical or chromatographic grade.

### 4.2. Modified Supercritical CO_2_ Extraction Procedure

A Spe-ed SFE-2 system (Applied Separation, Franklin, PA, USA) was used for all extraction, which operates with two pumps, a master pump for delivery of CO_2_ and second pump (Knauer pump, model K-501, Berlin, Germany) for the addition of modifier. An accurately weighed quantity of the grounded sample (about 0.5 g) was placed in a 10 mL volume extraction vessel (60 × 15 mm, i.d.) sandwiched with Celite. Before starting of extraction process the extraction vessel was preheated by oven for 10 min. The extraction conditions were as follows: pressure from 10 to 30 MPa; temperature from 40 to 60 °C; ethanol concentration from 60% to 90% (ethanol–water, *v*/*v*); flow-rate of CO_2_ (gaseous state), 2 L/min; flow-rate of modifier, 0.4 mL/min (correspond to 8% modifier). Inserting restrictor outlet into a vial containing ethanol collected extract. The collection vial was placed in an ice-water batch to aid trapping. The final volume of the extract was adjusted to 50.0 mL ethanol. This solution was utilized for HPLC detection. Dry products were removed solvents by rotary evaporation. Different concentrations of the products were made (25, 50, 100, 200 μg/mL) for DPPH radical scavenging activity assay and 3T3-L1 cell culture. 

### 4.3. HPLC Analysis

A high-performance liquid chromatography system (Hitachi, Tokyo, Japan) equipped with a Hitachi pump (model L-2130) and an ultraviolet-visible detector (Hitachi, model L-2400) was used. Analysis condition was as follows: Diamonsil C_18_ collumn (5 μm, 250 × 4.6 mm i.d., Dikma Technologies, Beijing, China); The mobile phase was A (acetonitrile) and B (0.2% phosphoric acid) in a step gradient manner (0–10 min, 15%–18% A; 10–30 min, 18%–23% A; 30–40 min, 23% –30%A; 40–50 min, 30%–40% A; 50–60 min, 40%–15%A) ; flow-rate, 1 mL/min; detection wavelength, 360 nm. 

Qualitative HPLC chromatography was performed on seven standards as follows: rutin, hyperin, isoquercetin, hibifolin, myricetin, quercetin-3′-*O*-glucoside and quercetin. Linear regression analysis for each of the seven standards was performed by plotting the peak area (Y) versus content (X, ng/mL). The linear calibration curves, linear ranges and correlation coefficients (R^2^) are shown in Table 4. The extraction yields of seven compounds for all experiments were calculated by the calibration curves. The sum of the contents of the seven pharmacologically active flavonoids was taken as the major flavonoid content.

### 4.4. DPPH Radical Scavenging Activity Assay

The DPPH radical assay has been widely used to evaluate the antioxidant activity of various active materials. DPPH radical scavenging activity was tested according to the method [31] with some modifications. Briefly, each extract was diluted with ethanol to form a series of concentration gradients. One hundred μL of each sample solution was added to 96- well plate, and then mixed with 100 μL of ethanol solution containing DPPH radicals (0.2 mmol/L). After incubation for 30 min at room temperature in the dark, the absorbance of reactants was measured at 517 nm in a Benchmark plus microplate spectrophotometer reader (Bio-Rad, Philadelphia, PA, USA). Antioxidant activity was expressed as an inhibition percent of DPPH radical and calculated from Equation (3):

(3) $$\text{DPPH radical scavenging activity }\left(\%\right)=\left[\frac{A_{negative\;control}-A_{sample}}{A_{negative\;control}}\right]\times 100\%$$

where $A_{negative\;control}$ was the absorbance of the control (without extract); $A_{sample}$ was the absorbance of the sample. Then the antioxidant activity was shown as the effective concentration at 50% (IC_50_ value), the concentration of sample required to scavenge 50% DPPH free radicals, were calculated by nonlinear regression analysis and expressed in mg/mL.

### 4.5. Box-Behnken Experimental Design

In this work, a Box-Behnken experimental design with three variables at three levels was used, which allows us to determine the interactions among the factors and improve the quality of prediction [22]. The software Design Expert (Stat-Ease Inc., Minneapolis, MN, USA) was employed for experimental design, model building and data analysis.

The effect of extraction pressure (X_1_), temperature (X_2_) and ethanol content (X_3_) on major flavonoids content (Y_1_) and IC_50_ value of antioxidant activity (Y_2_) was investigated using a Box-Behnken statistical model. The 17 runs of independent variables and responses are shown in Table 5. 

### 4.6. Statistical Analysis

A second-order polynomial model was used to evaluate the relationship between the response and the variables, which can be expressed as the following Equation (4):

(4) $$Y=\beta_{0}+\beta_{1}X_{1}+\beta_{2}X_{2}+\beta_{3}X_{3}+\beta_{11}X_{1}^{2}+\beta_{22}X_{2}^{2}+\beta_{33}X_{3}^{2}+\beta_{12}X_{1}X_{2}+\beta_{13}X_{1}X_{3}+\beta_{23}X_{2}X_{3}$$

where Y is the predicted response; X_1_ − X_3_ are independent factors that influence the response Y; β_0_ is a constant; β_1_ − β_3_ are the linear coefficients for the main variable effects, β_11_, β_22_ and β_33_ are quadratic coefficients, β_12_, β_13_ and β_23_ are interaction coefficients. Analysis of variance (ANOVA) was employed to analyze the chosen model and view the results. The statistical significant difference was defined as a probability (*p*-value) less than 0.05 and coefficient of determination (*R*^2^) greater than 0.900.

### 4.7. Soxhlet Extraction

A known quantity of ground sample (about 0.5 g) was accurately weighed into an extraction thimble. Extractions were carried out in a Soxhlet extractor with 50 mL of 95% ethanol in an 87 °C water bath. After 28 cycles in the Soxhlet equipment, the extracts were transferred to a 50 mL volumetric flask and made up to the mark with 95% ethanol.

### 4.8. 3T3-L1 Cell Culture and Adipocyte Differentiation

In 6-well plates, 3T3-L1 preadipocytes were grown in DMEM supplemented with 10% FBS and antibiotics (100 units/mL penicillin and 100 g/mL streptomycin). The culture was maintained in a 5% CO_2_ atmosphere at 37 °C. Two days post-confluence, 3T3-L1 cells (designated as day 0) were stimulated by a differentiation mixture culture containing 1 μM DEX, 0.5 mM IBMX and 1 μg/mL insulin in DMEM with 10% FBS for 3 days (days 0–2). Then cells were incubated in the medium with DMEM containing 10% FBS and 10 μg/mL insulin for 2 days further (days 3–4), and thereafter incubated in DMEM containing 10% FBS and refreshed at 2 day intervals (days 5–8). Dosage groups: adding the final concentration of 25, 50, 100 and 200 μg/mL extracts in the above cell culture fluid from day 0 to 8. Pioglitazone groups were given 10 μmol/L pioglitazone in the whole cell culture process.

### 4.9. Real-Time PCR Detection Cell Differentiation Marker Genes

Total RNA was extracted from the 3T3-L1 cells by an Rneasy MiNi Kit from the normal and dosage and pioglitazone groups on days 2, 4 and 8, and reverse transcription and real-time PCR were performed by a RT PrimeScript Kit and SYBR Premix Ex Taq, respectively. Table 6 shows the primer sequences that were synthesized by Sangon Biotech Co., Ltd. (Shanghai, China). To enable comparison of Ct (cycle threshold) values between groups, quantities of all target genes in the test samples were normalized by GAPHD. The results of relative mRNA expression were used in formula:
2^−△△Ct^, △Ct = Ct value (target gene) − Ct value (GAPHD), △△Ct = △Ct (dosage (5)group) − △Ct (control group)


All results obtained were expressed as mean ± SD. Statistical analysis was performed using the SPSS software (22.0 version; IBM, Armonk, NY, USA). Significant differences (*p* < 0.05) were determined by One-way ANOVA.

## 5. Conclusions

In this study, the optimized SFE extraction parameters were screened by a Box-Behnken response surface methodology experiment. This is the first study on utilization of modified superficial CO_2_ for the extraction of flavonoids from the flowers of *A. manihot*, quantitative analysis by HPLC and antioxidant activity determination by IC_50_ value. The optimum parameters for extraction of flavonoids were 20 MPa, 53 °C,and 85% ethanol content. The results suggest that the flowers of *A. manihot* can be a good source of natural antioxidants and SFE is an efficient method to extract its flavonoids. Furthermore, the extracts might inhibit adipogenic activity through down-regulation of the expression of PPARγ and C/EBPα (major adipogenic transcription activator) at mRNA in 3T3-L1 adipocytes. Therefore, flavonoids may provide a possible therapeutic approach for the prevention and treatment of obesity.

## Figures and Tables

**Table 1:** ANOVA for the fitted quadratic polynomial model for major flavonol content.

Source	Sum of Squares	Degrees of Feedom	*F*	*P*	*R^2^*	*R^2^ (Adj)*
Model	163.93	9	71.18	<0.0001	0.9892	0.9753
X_1_	1.74	1	6.80	0.0351		
X_2_	17.79	1	69.52	<0.0001		
X_3_	58.54	1	228.74	<0.0001		
X_1_ X_2_	0.28	1	1.10	0.3296		
X_1_ X_3_	0.17	1	0.67	0.4390		
X_2_ X_3_	1.38	1	5.39	0.0532		
X_1_^2^	37.20	1	145.38	<0.0001		
X_2_^2^	36.21	1	141.49	<0.0001		
X_3_^2^	3.53	1	13.77	0.0075		
Residual	1.79	7				
Lack of fit	1.32	3	3.70	0.1191		
Pure error	0.47	4				
Cor total	165.73	16				

**Table 2:** ANOVA for the fitted quadratic polynomial model for IC_50_ value of antioxidant activity.

Source	Sum of Squares	Degrees of Feedom	*F*	*P*	*R^2^*	*R^2^ (Adj)*
Model	0.04	9	13.51	0.0012	0.9456	0.8756
X_1_	0.0090	1	27.22	0.0012		
X_2_	<0.0001	1	0.024	0.8806		
X_3_	0.017	1	53.02	0.0002		
X_1_ X_2_	0.0011	1	3.03	0.1120		
X_1_ X_3_	0.0037	1	11.28	0.0121		
X_2_ X_3_	0.0005	1	1.60	0.2458		
X_1_^2^	0.0011	1	3.27	0.1136		
X_2_^2^	0.0027	1	8.30	0.0236		
X_3_^2^	0.0037	1	11.11	0.0125		
Residual	0.0023	7				
Lack of fit	0.0014	3	2.03	0.2517		
Pure error	0.0009	4				
Cor total	0.042	16				

**Table 3:** Comparison of the dependent variables obtained by SFE and Soxhlet extraction.

Values	Dependent Variables *	Method	
		SFE	Soxhlet
Predicted value	Y_1_ (mg/g)	41.96	-
	Y_2_ (mg/mL)	0.288	-
Experimental value	Y_1_ (mg/g)	41.58 ± 1.16	40.69 ± 2.07
	Y_2_ (mg/mL)	0.287 ± 0.008	0.281 ± 0.009

* mean ± SD, *n* = 3.

**Table 4:** Linear calibration curves, linear ranges and correlation coefficients for the seven flavonoids.

Compound	Linear Calibration	Linear Range	*R^2^*
Rutin	Y = 32393X + 4828.5	0.3–125.0	0.9998
Hyperin	Y = 38981X − 19675	3.7–150.0	0.9999
Isoquercetin	Y = 35082X + 259.2	2.2–111.0	1.0000
Hibifolin	Y = 46777X + 3563.3	5.0–250.0	0.9999
Myricetin	Y = 39487X − 6277.1	1.1–50.0	0.9998
Quercetin-3*′-O*-glucoside	Y = 71346X − 631.5	5.0–250.0	0.9999
Quercetin	Y = 78474X − 742.2	3.1–50.6	0.9999

**Table 5:** The Box-Behnken experimental design and experimental variables (X_1_: pressure; X_2_: temperature; X_3_: ethanol content) and responses (Y_1_: major flavonol content; Y_2_: IC_50_ value).

Run	X_1_ (MPa)	X_2_ (°C)	X_3_ (*V_ethanol_*/*V_water_*, %)	Y_1_ (mg/g)	Y_2_ (mg/mL)
1	30	50	60	34.79	0.447
2	30	40	75	33.56	0.357
3	20	40	60	32.48	0.435
4	20	50	75	40.33	0.305
5	30	60	75	36.11	0.407
6	20	50	75	39.92	0.319
7	20	50	75	40.51	0.296
8	20	60	60	34.19	0.391
9	20	50	75	39.97	0.314
10	10	40	75	32.13	0.315
11	20	40	90	37.52	0.302
12	20	50	75	40.72	0.281
13	10	60	75	35.74	0.299
14	20	60	90	41.58	0.304
15	10	50	90	38.43	0.311
16	30	50	90	38.98	0.309
17	10	50	60	33.41	0.327

**Table 6:** Primer sequences used for real-time PCR.

Mouse Primers	Forward Primer	Reverse Primer
PPARγ	TTCAGAAGTGCCTGGCTGTG	TCTTTCCTGTCAAGATCGCC
C/EBPα	AGGAACACGAAGCACGATCAG	CGCACATTCACATTGCACAA
GAPHD	AATGACCCCTTCATTGAC	TCCACGACGTACTCAGCGC

## Supplementary Materials

Supplementary materials can be accessed at: http://www.mdpi.com/1420-3049/21/7/810/s1.
