# Supplementary material for: Extraction of Flavonoids from the Flowers of Abelmoschus manihot (L.) Medic by Modified Supercritical CO2 Extraction and Determination of Antioxidant and Anti-Adipogenic Activity
Source: Molecules. 2016 Jun 25;21(7):810. doi: 10.3390/molecules21070810 (PMC6273967; doi:10.3390/molecules21070810)
Supplement: Supplementary file 1 [file molecules-21-00810-s001.pdf]

Supplementary Materials: Extraction of Flavonoids from the Flowers of *Abelmoschus manihot* (L.) Medic by Modified Supercritical CO_2 Extraction and Determination of Antioxidant Activity and Anti-Adipogenic Activity

Jingjing Li, Juan Zhang and Min Wang

1. Establishment of Extraction Parameters by Single Factor Evaluation

The range of extraction times, temperatures, modifier constituents and pressure was investigated by previous single-factor experiments before the Box-Behnken experimental design was performed.

1.1. Extraction Pressure

The effects of extraction pressure on the content of major flavones were investigated, while the others extractive conditions were time 2 h, temperature 50 °C and 80% ethanol content (Figure S1).

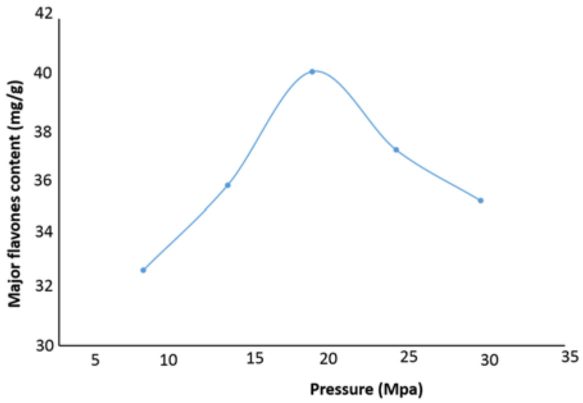

Figure S1. The effects of extraction pressure on the major flavones' content.

1.2. Extraction Temperature

The effects of extraction temperature on the content of major flavones were investigated, while the other extractive conditions were time 2 h, pressure 20 MPa and 80% ethanol content (Figure S2).

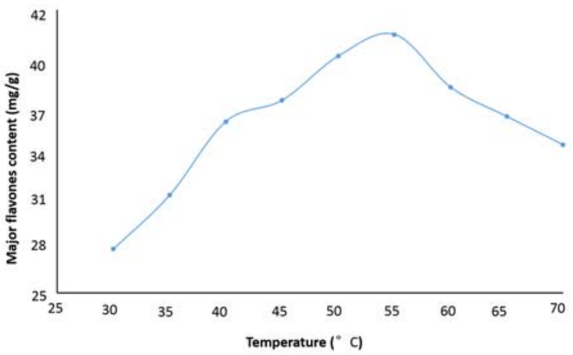

Figure S2. The effects of extraction temperature on the major flavones' content.

1.3. Ethanol Content

The effects of ethanol content on the content of major flavones was investigated, while the other extractive conditions were time 2 h, pressure 20 MPa and temperature 50 °C (Figure S3).

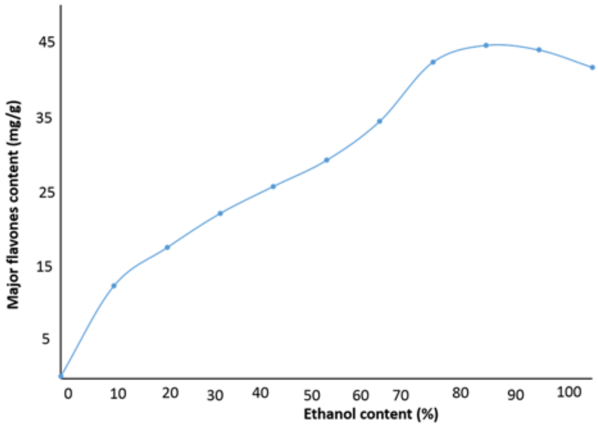

Figure S3. The effects of extraction ethanol content on the major flavones' content.

2. The Constituents of Extracts in HPLC Chromatograms

Comparing the constituents of extracts and seven flavonoid standards by HPLC, we identified the major flavonoid components as follows: rutin, hyperin, isoquercetin, hibifolin, myricetin, quercetin-3′-O-glucoside and quercetin (Figure S4).

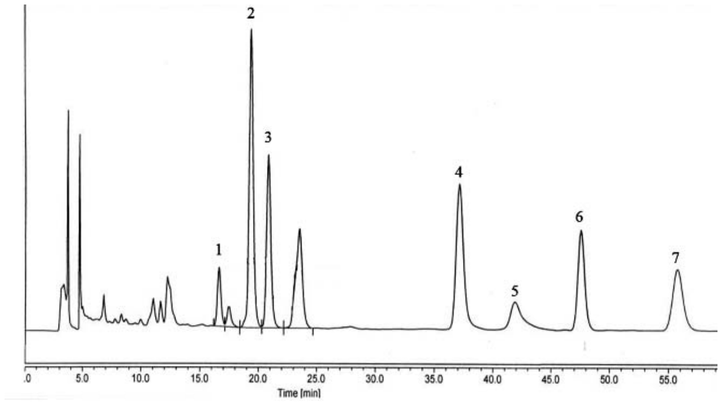

Figure S4. The HPLC chromatograms of extracts. [1] rutin; [2] hyperin; [3] isoquercetin; [4] hibifolin; [5] myricetin; [6] quercetin-3′-*O*-glucoside; [7] quercetin.

3. The Effect of Extraction Parameters

3.1. The Effect of Temperature and Pressure on Major Flavones' Yields

It was observed that higher major flavones yield (> 40 mg/g) was attained when the pressure was set between 18 MPa to 25 MPa and temperature between 48 °C to 57 °C (Figure S5)

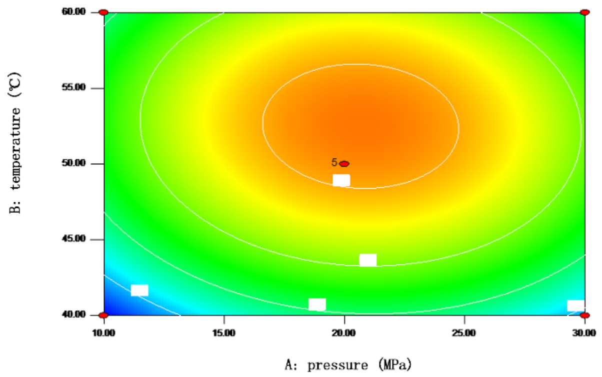

Figure S5. The effect of pressure and temperature on major flavones content at constant 75% ethanol concentration.

3.2. The Effect of Temperature and Pressure on IC_{50}

The lower value of IC_{50} (<0.300 mg/mL) occurred at pressures below 18 MPa and temperatures above 45 °C

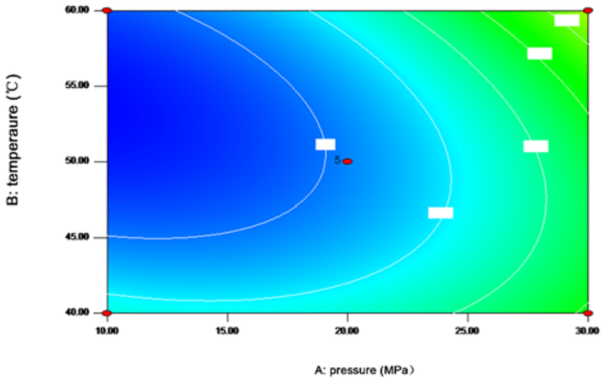

Figure S6. The effect of pressure and temperature on IC_{50} at constant 75% ethanol concentration.

3.3. The Effect of Pressure and Ethanol Content on Major Flavone Content

A higher major flavones content (>40.0 mg/g) was obtained at pressures between 18 MPa and 23 MPa and ethanol concentrations above 80% (Figure S7)

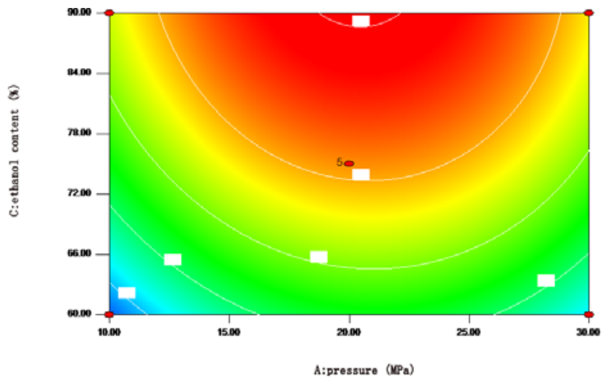

Figure S7. The effect of pressure and ethanol content on major flavones content at constant temperature 50 °C.

3.4. The Effect of Pressure and Ethanol Content on IC_{50}

A lower IC_{50} value (<0.300 mg/mL) of extracts was obtained when the pressure was less than 20 MPa and the ethanol concentration ranged from 75% to 85% (Figure S8).

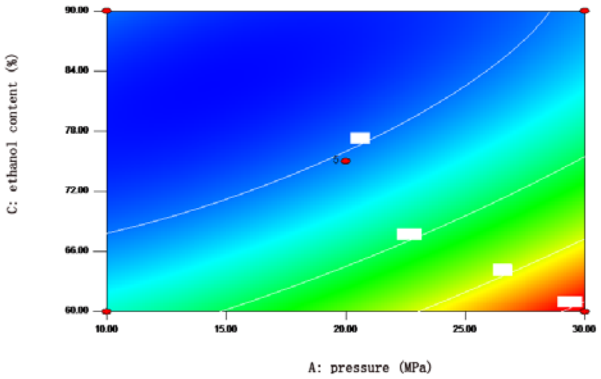

Figure S8. The effect of pressure and ethanol content on IC_{50} at constant temperature 50 °C.

3.5. The Effect of Temperature and Ethanol Content on Major Flavone Content

When considering the effects of temperature and ethanol content on the flavone content, higher yields were obtained at higher temperature and higher ethanol content (Figure S9).

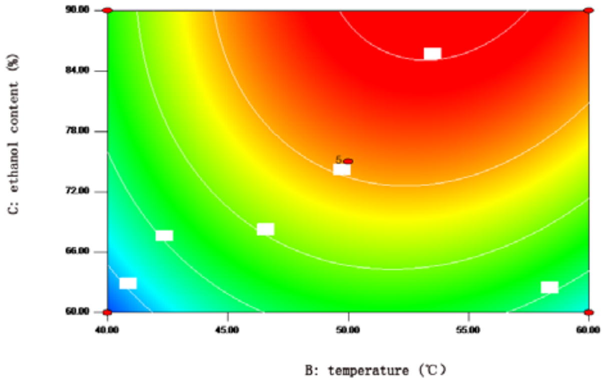

Figure S9. The effect of temperature and ethanol content on major flavones content at constant pressure 20 MPa.

3.6. The Effect of Pressure and Ethanol Content on IC_{50}

For the DPPH radical-scavenging activity of the extracts, smaller values of IC_{50} (<0.300 mg/mL) were attained when the ethanol content was set above 78% and the temperature below 55 °C (Figure S10).

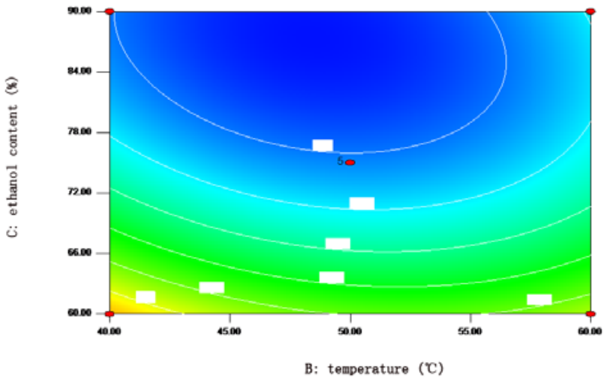

Figure S10. The effect of pressure and ethanol content on IC_{50} at a constant pressure of 20 MPa.
